# ‘Once there is life, there is hope’ Ebola survivors' experiences, behaviours and attitudes in Sierra Leone, 2015

**DOI:** 10.1136/bmjgh-2016-000108

**Published:** 2016-11-18

**Authors:** Emilie Karafillakis, Mohamed F Jalloh, Azizeh Nuriddin, Heidi J Larson, Jimmy Whitworth, Shelley Lees, Kathy M Hageman, Paul Sengeh, Mohammad B Jalloh, Rebecca Bunnell, Dianna D Carroll, Oliver Morgan

**Affiliations:** 1 Department of Infectious Disease Epidemiology, London School of Hygiene & Tropical Medicine, London, UK; 2 FOCUS 1000, Freetown, Sierra Leone; 3 US Centers for Disease Control and Prevention, Atlanta, Georgia, USA; 4 Department of Global Health and Development, London School of Hygiene & Tropical Medicine, London, UK; 5 Division of Global Health Protection, US Centers for Disease Control and Prevention, Atlanta, Georgia, USA; 6 Department of Research and Evaluation, FOCUS 1000, Freetown, Sierra Leone; 7 National Center on Birth Defects and Developmental Disabilities, US Centers for Disease Control and Prevention, Atlanta, Georgia, USA

## Abstract

**Background:**

In Sierra Leone, over 4000 individuals survived Ebola since the outbreak began in 2014. Because Ebola survivorship was largely unprecedented prior to this outbreak, little is known about survivor experiences during and post illness.

**Methods:**

To assess survivors' experiences and attitudes related to Ebola, 28 in-depth interviews and short quantitative surveys with survivors from all four geographic regions of Sierra Leone were conducted in May 2015.

**Results:**

Survivor experiences, emotions and attitudes changed over time as they moved from disease onset to treatment, discharge and life post-discharge. Survivors mentioned experiencing acute fear and depression when they fell ill. Only half reported positive experiences in holding centres but nearly all were positive about their treatment centre experiences. Survivor euphoria on discharge was followed by concerns about their financial situation and future. While all reported supportive attitudes from family members, about a third described discrimination and stigma from their communities. Over a third became unemployed, especially those previously engaged in petty trade. Survivor knowledge about sexual transmission risk reflected counselling messages. Many expressed altruistic motivations for abstinence or condom use. In addition, survivors were strongly motivated to help end Ebola and to improve the healthcare system. Key recommendations from survivors included improved counselling in holding centres and long-term government support for survivors, including opportunities for participation in Ebola response efforts.

**Conclusions:**

Survivors face myriad economic, social and health challenges. Addressing survivor concerns, including the discrimination they face, could facilitate their reintegration into communities and their contributions to future Ebola responses.

**Key questions**

**What is already known about this topic?**

- There is currently little information available about the experiences, behaviours and attitudes of Ebola survivors.
- The latest West-African Ebola outbreak led to an unprecedented number of Ebola survivors, who have been shown to suffer from physical and mental sequelae.
- Studies have now started to investigate the extent and characteristics of those Ebola complications and a few studies have also identified that Ebola survivors may face discrimination and stigmatisation from their communities and their loved ones.

**What are the new findings?**

- Owing to the recent nature of the outbreak, there is still a lot unknown about the experiences, behaviours and attitudes of Ebola survivors.
- This study addresses those issues in Sierra Leone and aims to improve our understanding of survivors' experiences during and after Ebola infection.
- It extends our knowledge of issues that have not yet been discussed extensively such as community acceptance of survivors, attitudes towards sexual transmission or the role survivors can play in Ebola outbreak control activities.

**Recommendations for policy**

- The results from this study will inform responses to future Ebola outbreaks by increasing our understanding of the patient experience and challenges faced by survivors.
- By understanding the experience of persons infected with Ebola virus, epidemic control measures can be adapted to reduce mistrust and increase cooperation with affected communities.

## Introduction

The Ebola outbreak that began in 2013 in West Africa was the largest in recorded history. The region faced many challenges in addressing the emerging infectious disease. Contributors to the rapid spread of Ebola included poverty, poor healthcare systems and services, inadequate surveillance systems, community distrust of outbreak response teams and contact with infected people at home, in healthcare facilities and during traditional burials.^1^ ^2^ Identifying and addressing the knowledge, attitudes and practices of the public played a key role in addressing some of these contributors.

In Sierra Leone, the outbreak was declared in May^3^ and peaked in November 2014,^4^ but the outbreak response varied from one district to the next, as each district faced the disease at different times and intensities.^5^ Though mortality associated with this outbreak was high, there have never been so many Ebola survivors before. In Sierra Leone alone, there were over 14 000 cases with an estimated 4051 survivors.^6^ Many survivors are suffering from physical and mental sequelae.^7^ ‘Post-Ebola virus disease syndrome’ characterises these complex symptoms, which may affect survivors' daily functions and ability to work and may include chronic joint and muscle pain, fatigue, hearing and vision loss, depression and memory problems.^8^ ^9^

Surveys have also identified that Ebola survivors may face discrimination and stigmatisation from their communities and their loved ones. In August 2014, a national household survey conducted in Sierra Leone showed that 96% of respondents held at least one discriminatory attitude towards Ebola survivors.^10^ Although declining, 46% of respondents still held at least one discriminatory viewpoint in July 2015.^11–14^ In Sierra Leone, The Comprehensive Program for Ebola Survivors (CPES) has been developed to protect Ebola survivors. The WHO, together with partner organisations involved in the Ebola response, offered technical support for the development and implementation of this program. It aims to ensure Ebola survivors access the health and social welfare services they require. It includes programs that target stigmatisation and the health-related complications of the disease, and also aims to prevent further transmission, especially through sexual intercourse. It includes counselling and access to health services such as eye clinics, sexual health counselling for individuals and couples, and semen testing.^15^ ^16^

In April–May 2015, a qualitative evaluation was conducted to assess survivors' knowledge, attitudes, practices and experiences related to Ebola in Sierra Leone in order to understand survivor needs and stigma directed towards them. These findings may help inform national and local efforts to strengthen services for survivors, address identified barriers and reinforce trust in the healthcare system in a post-Ebola environment in Sierra Leone.

## Methods

This evaluation followed a mixed method design consisting of qualitative interviews followed by short post-interview surveys. The post-interview survey was conducted in order to quantify key thematic areas from the in-depth interviews (IDIs). Triangulating the short survey data with the in-depth qualitative findings aimed to provide a more accurate narrative of survivors' experiences, perceptions, attitudes and practices.

### Sampling and selection of participants

The evaluation consisted of 28 IDIs with survivors at least 18 years of age, followed by a short survey conducted immediately after each IDI. The researchers discussed data saturation and estimated it to be reached at or after 20 interviews. Five districts of Sierra Leone were purposively selected to cover all four geographic regions of the country and to include survivors with varied Ebola Treatment Units (ETUs) discharge dates: Western Urban and Western Rural (both western area), Kambia and Port Loko (both northern province), Kono (eastern province) and Moyamba (southern province). Four interviews were conducted in each district, except in Kambia, where eight individuals participated to include survivors from the Guinea border area. Survivors from Guinea were excluded and only those who were infected in Kambia and were treated in an ETU in Kambia or Port Loko were included in the study. ETUs' psychosocial counsellors and Survivor Psychosocial Networks present in each district were contacted to purposively identify survivors which were interviewed in their homes alone. Snowball sampling was also used to ask participants to refer other potential Ebola survivors. Diversity in participants' sex, discharge date, rural versus urban setting and age was ensured in the selection process. No relationship with participants was established prior to study initiation, and all participants provided informed consent for their participation in this study. Participants had to be ≥18 years old and had to present an Ebola survivor certificate issued by the government of Sierra Leone. No participant refused to participate or dropped out of the study.

### Data collection

Data collection occurred between April and May 2015. Facilitators and note-takers, both males and females, were trained during a 3-day workshop on how to conduct the IDIs. They were led by a senior program manager of FOCUS1000 (MFJ). The interview guide and protocols were first developed by two behavioural scientists with field experience of working in the Ebola response in Sierra Leone. The draft instruments were then reviewed by a senior scientist with extensive experience working with HIV populations in sub-Saharan Africa. Local contextualisation and cultural appropriateness were ensured by a second review from a partner from a local non-governmental organisation (NGO) with experience in conducting knowledge, attitude and practice (KAP) assessments in Sierra Leone. The instruments were then pre-tested by the behavioural scientists with three survivors from the Western Area (which were not included in the final study results) and feedback was used to revise and finalise the instruments. Each IDI (see online supplementary appendix 1) was directly followed by a post-interview survey (see online supplementary appendix 2), which was administered face-to-face by the IDI facilitator using a paper-based instrument. Interviews were conducted in Krio, the most widely spoken language in Sierra Leone, to ensure consistency of the interview questions and to help standardise questions and probes. However, some keywords were translated to the dominant local languages and participants were given the opportunity to express their views on particular subjects in their local languages if necessary. All survivors interviewed were fluent and agreed to be interviewed in Krio. Interviews were tape-recorded and supplemented with hand-written notes. Facilitators and note-takers transcribed and translated the recordings into English. All transcripts were reviewed by a separate supervisory team to ensure accuracy.

10.1136/bmjgh-2016-000108.supp1 Supplementary data

### Coding and data analysis

Through a deductive coding process, two of the authors developed and reached consensus on parent codes drawn from the IDI guide. Subcodes were developed using an inductive process. Evaluators coded transcripts using Dedoose, a web-based, qualitative analysis software package and achieved consensus on a final codebook which was used to code all transcripts. Key themes were then identified and summarised. The methodological orientation underpinning the study was a phenomenological inquiry. No repeat interviews could be carried out, and transcripts and findings could not be returned to participants for feedback due to the ongoing Ebola outbreak at the time of the interviews. However, after each interview, major points discussed were repeated to participants for concurrence.

Post-interview survey data were entered into Open Data Kit (ODK) and subsequently uploaded to Kobo Collect's web-server for aggregation. The data repository was then imported into SPSS V.22 for analysis. The analysis included the generation of descriptive statistics using frequency tables as well as cross-tabulations using contingency tables.

### Ethical approval

The Sierra Leone Ethics and Scientific Review Committee approved the protocol and the US Centre for Disease Control (CDC) Institutional Review Board (IRB) determined the project to be public health non-research. This indicates data collection with human participants was done as a routine public health practice in the context of an unprecedented epidemic and, therefore, does not require ethical approval.

## Results

### Participant characteristics

Participants (table 1) included 14 males and 14 females, with a mean age of 31 years (ranging from 18 to 67 years). Survivors were discharged from ETU an average of 4 months (range: 2–7) prior to the interviews, indicating that many were infected and treated around the outbreak peak (November 2014^4^). Unemployment among participants rose from 7.1% prior to Ebola infection to 39.3% at the time of the interviews with a decrease in the number of private business owners (from 10.7% to 3.6%), petty traders (from 32.1% to 10.7%), students (from 10.7% to 7.1%) and teachers (from 7.1% to 3.6%), and an increase in the number of medical or health professionals (from 10.7% to 14.3%) and government employees (from 0% to 7.1%). The increase in the number of health professionals was due to the recruitment and involvement of some Ebola survivors in Ebola control activities.

**Table 1:** Demographic characteristics of interviewed Ebola survivors, Sierra Leone, April–May 2015 (N=28)

	Total (#)
Location of interview	
Western Urban	4
Western Rural	4
Kambia	8
Moyamba	4
Port Loko	4
Kono	4
Age, years	
18–25	10
26–64	16
≥65	2
Marital status (at time of interview)	
Single	19
Married or with a partner	9
Number of months since release from ETUs (months)	
2	6
3	5
4	5
5	6
6	5
7	1
Location at time of illness	
Same district as where interview was conducted	25
Different district as where interview was conducted	3
Education level	
No formal education	7
Some primary school	3
Completed primary school	2
Completed Junior secondary school	8
Completed Senior secondary school	4
Completed post-secondary education or training	4
Employment at time of interview	
Private business	1
Plumber/carpenter/electrician	0
Petty Trader	3
Farmer	3
Teacher/lecturer/instructor	1
Motorcycle taxi driver	1
Medical or health professional	4
Student	2
Other government employee	2
Unemployed	11

### Survey results

The survey found that 25% (7 of 28) of the survivors interviewed (42.9% males vs 57.1% females) moved home after their return from ETUs and 42.8% (3/7) of those moved to a new district. 89.3% (25 of 28) lived with their relatives at the time of the interviews, of which 88% (22 of 25) had lived with the same people before getting sick.

When questioned about potential sources of transmission, 60.7% (17 of 28) of participants stated that they had taken care of sick individuals (58.8%—10 of 17—of which were females), 46.4% (13 of 28) were living in the same household as someone suspected or confirmed to have Ebola, 14.3% (4 of 28) had participated in a burial or funeral (75%—3 of 4—of which were males, all released 6–7 months prior to the interviews) and 7.1% (2 of 28) were unsure of their source of infection. 61.5% (8 of 12) of those released 5–7 months prior to the interviews had shared a household with someone suffering from Ebola compared to 38.5% (5 of 16) of those released 2–4 months prior to the interviews.

75% (21 of 28) of survivors interviewed first sought treatment from a medical professional at a healthcare facility (61.8%—13 of 21—of which were released from an ETU 2–4 months prior to the interview), with only 21.4% (6 of 28) of participants having sought treatment from a medical professional outside a healthcare facility (66.6%—4 of 6—of which were released 5–6 months prior to the interview) and 3.6% (1/28) from a traditional leader (released 4 months prior to the interview). The mean number of days it took for participants to seek treatment after feeling sick was 3.36 (SD 1.7).

Participants described their day-to-day interaction with community members since their release from an ETU as very good (13 of 28, 46.4%), good (9 of 28, 32.1%), not good (4 of 28, 14.3%) or very bad (2 of 28, 7.1%). More women than men had negative experiences, with 28.5% (4 of 14) of women describing a negative community interaction compared to 14.2% (2 of 14) of men. Furthermore, 66.6% (4 of 6) of those who described a negative experience and 54.5% (12 of 22) of those who described a positive experience were released from an ETU 2–4 months prior to the interview.

A minority of survivors interviewed (8 of 28, 28.6%) stated that they had engaged in sexual intercourse since their ETU discharge (50% of which were females), with 50% (4 of 28) of them also reporting not having used a condom during their last intercourse (75% of which were females). The results showed that 87.5% (7 of 8) of those sexually active and 75% (3 of 4) of those who did not use a condom were released from an ETU 5–7 months prior to the interview.

### Survivors' emotional and physical state regarding illness

On diagnosis, nearly all survivors recalled feeling terrified about the uncertainty of what would happen to them, and being sick with Ebola made them feel depressed. A few survivors also acknowledged feeling shocked, as they had initially believed Ebola did not exist in Sierra Leone: “I never believed Ebola was real, so I was emotionally confused. (…) I thought nothing was wrong with me” (Moyamba, female). After learning of their diagnosis, most survivors thought death was inevitable, while some felt they were already dead. Having seen others die from Ebola, the prospect of a similar fate was reported to be emotionally challenging. They also explained they were afraid of changes in their physical appearance. Many reported being extremely weak and unable to stand, speak, sit, walk or work; they felt helpless and tormented.

### Beliefs on how infection occurred

Most survivors believed they contracted the disease by helping or caring for a sick family member: “I believe I got infected when I was caring for my sick wife. When she was sick, she couldn't do anything on her own (…). I used to clean her and helped her when she wanted to do anything” (Western Area Rural, male). Some also discussed attending burials or funerals before contracting the disease. One survivor stated: “After the burial, mourners and other people within the compound got sick and started showing signs [of Ebola]. I, as one of them, also fell sick” (Kambia, female).

### Beliefs and experience with care seeking

As national response coordination consolidated, individuals were instructed to report suspected Ebola cases to the national 117 telephone hotline or local call numbers. Before alerting health officials, many survivors tried self-medication such as oral-rehydration therapy, a common practice used for cholera and other endemic diarrheal diseases. A few survivors also described calling the 117 helpline or informing community leaders to report illness as a mean of survival. One survivor described first seeking care from a traditional healer. Survivors reported their family, community members or sometimes staff working in a quarantined area calling 117 for them, without giving them a choice: “Since I was under quarantine, there was no way to seek help elsewhere” (Western Area Rural, male).

Survivors described varied experiences with the ambulances that took them to the Ebola Holding Units (EHU). Many reported being afraid, especially of the smell and use of chlorine to disinfect the vehicles. A woman from Western Area also explained: “[I] went straight to the Ambulance but when I touched the door, one of the ambulance guys shouted at me and sprayed where I touched the door. I was not happy.” A minority of survivors also expressed dissatisfaction with the ambulance services, because the ambulances were reportedly too hot and had locked windows. Some were scared of the health workers' personal protective outfits, or of the negative image associated with the ambulances: “My mother and sisters were all crying bitterly, falling on the ground and giving remarks that I will die if I enter the Ambulance” (Kono, female). However, many survivors talked positively about the ambulance services, and praised the health workers who “started counselling me, saying that I should not be afraid and I will be ok and nothing is going to happen to me” (Western Urban, female).

### Experience with the EHU and ETU

Following an initial assessment, patients were generally transported to EHUs, where blood samples were tested. If positive, patients would be transferred to an ETU. Ideally, results were given within 24 hours, but early in the response this process was sometimes delayed by several days. While about half of survivors reported positive experiences at the EHUs and felt that they were well taken care of; the other half described EHU staff as unkind, inattentive and refusing to be in close physical contact with patients. In retrospect, a few survivors believed staff at EHUs lacked knowledge of Ebola because they told them there was no treatment available to cure the disease and yet they survived. Some survivors reported that they did not receive counselling, did not have enough food or drugs, and were left alone in an unsafe and untidy environment. One survivor mentioned: “Because of the poor treatment by the doctor I was afraid to eat the food they served me in the holding centre. I thought they wanted to kill me” (Kambia, female).

Individuals who tested positive for Ebola at the EHUs were transferred to an ETU. Nearly all interviewed survivors commented that ETU staff was kind and supportive and they were ‘treated like kings’. Survivors described being pleased with the free food and medication, and appreciated counselling from health workers, including international staff. They frequently expressed enjoying the company of polite and friendly facility staff. They also mentioned being treated fairly and equally, independently of their ‘race, colour or tribe’.

### Emotional state after discharge from the ETUs

Most survivors explained they were relieved after being discharged from the ETUs, felt like ‘heroes’, and thanked God for their survival. Some saw their recovery as a new start to life: “Once there is life, there is hope: a dead man cannot work, a dead man cannot move” (Western Area Rural, male). However, feelings of sadness, depression and anger were also expressed by a lot of survivors and they reported feeling unsupported. “Since I was discharged I have been crying for help but nobody helps me” (Western Areal Rural, female). Some survivors also expressed frustration after being discharged because their everyday life had deteriorated: “There are times I have the feeling that life is worthless as I now don't engage in anything and now some community people are neglecting me” (Port Loko, male). A few also reported feelings of loneliness and alienation due to loosing family members to the disease.

### Family relationships

Most survivors described living ‘happily’, and having a good relationship with their families before becoming infected with Ebola. They further shared that such relationships did not change after their return from the ETUs, even though some households were grieving loss of other family members who had died. All interviewed survivors reported that their families welcomed them back with joy, and treated them well. Additionally, families took the roles of carers for interviewed survivors, providing either health, financial or housing support. Their lives were described, once again, as ‘normal’, as if nothing had changed: “My brothers are very much happy to see me back and do things together as we used to do” (Western Urban, male).

### Partner relationships

Several survivors who had a partner (boyfriends/girlfriends or husbands/wives) at the time of the interviews (9 of 28) did not report any major changes in their relationships. Only a few survivors mentioned that their partners, mostly boyfriends or girlfriends, did not treat them well when they returned from the ETUs or were afraid of or embarrassed by them. “Since the day [my boyfriend] knew I was Ebola positive, he stopped picking up my calls. (…) I know he was afraid or ashamed of me because I was infected with Ebola” (Kambia, female). While some partners ended their relationship with interviewed survivors, others reunited after ‘counselling sessions’ with social work staff.

### Sexual behaviour and knowledge of potential risks of Ebola sexual transmission

At the time of the interviews, messaging on sexual transmission of Ebola recommended abstinence or condom use out of caution as it was unknown whether Ebola could be sexually transmitted. All survivors reported being counselled to not have unprotected sexual intercourse for the first 90 days on discharge from the ETU, which was consistent with WHO interim advice given to survivors prior to May 2015. Many survivors conveyed that it would be risky for their partners to have sexual relationships with them. They overwhelmingly emphasised that they do not want others to go through what they had suffered. Some of the survivors reported that they decided to wait longer than the recommended 90 days and used condoms to make sure that their partner(s) would not be exposed to Ebola through sexual transmission.

In one instance, a male survivor from Port Loko expressed that all Ebola survivors should be quarantined for 3 months ‘so they will not be infecting people with the disease through their sexual activities’. Other survivors mentioned that they advised their community members not to have sexual intercourse with survivors. A handful of survivors expressed that this was mostly important for males or for people who have sex with multiple partners. Finally, a few survivors experienced a loss of libido and some women felt guilty about not having sex with their husbands. One male survivor also mentioned: “My wife denied me sometimes because of the fear of the disease” (Moyamba, male).

### Community relationships

The potential negative reaction of community members—sometimes including loved ones—emerged as a major concern for interviewed survivors when they initially suspected Ebola, which led some to deny feeling ill. However, the relationships between survivors and their communities were described positively both before and after Ebola by the majority of survivors. Survivors described themselves as social individuals, who were interactive and good ‘religious’ members of their communities, attending either the church or the mosque: “I am a Muslim observing my prayers. I have been a very good social person mingling with people in my community” (Kambia, female)*.* After they were discharged from ETUs, many survivors mentioned they were welcomed back into their communities and continued good relationships and interactions with community members, including religious and local leaders: “People living in my community have no problem with me as we interact together, attend community meetings, play games together and do things together” (Port Loko, male). Survivors shared that community members expressed their sympathy, especially if they had also lost members of their families and prayed for them.

However, a few survivors also experienced discrimination from members of their communities. They felt people were afraid of them, provoked them and stigmatised them as survivors: “Most of my neighbours abandoned and refused to accept me” (Kono, male). They were deeply affected by the discriminatory attitudes of their community, and felt abandoned, stressed and lonely due to provocations and stigmatisation. Some survivors also described being evicted from their houses by landlords who were scared of possible contagion. Some had reduced their interactions at the church or mosque and others stated that people actively avoided them and their children.

### Survivors' financial and employment situation

Survivors described their lives before becoming infected with Ebola as normal, comfortable, doing good business. However, most survivors reported losing their jobs, facing financial difficulties and being unable to take care of their families: “Before, I was employed but now, I am not employed. (…) The more you earn, the more you live well with your family” (Western Area Rural, male). Reasons for inability to work were lack of strength and lack of finance to continue or start their businesses. Most of those shifting to unemployment were previously engaged in petty trade and business. After discharge from the ETU, one survivor became a healthcare worker and another one a government official.

Other problems mentioned were difficulties paying for their children's school fees and feeling dependent on others: “'I find it difficult now to take care of my children because I have no money, no business (…). I have to beg for our survival, but how long would that continue for?” (Port Loko, male). Moreover, although most survivors reported having received financial benefits from various organisations after being discharged from the ETUs, some reported having received insufficient assistance to help them become financially independent.

### Improving the situation of survivors

Almost all survivors suggested that the government of Sierra Leone should help them by providing jobs, microcredit or training so they could develop necessary skills for employment. They also discussed the need for financial help and their desire to receive money, scholarships and other incentives. They mentioned their need for livelihood support, including the provision of food and supplies as well as housing. Finally, some survivors stated that the government should help in engaging communities and households on the discrimination of survivors. A woman from Moyamba explained that it is important “to sensitise community members (…) on the issue of discriminating Ebola survivors.” Another survivor said that the “government should pass laws in the parliament that no one should discriminate [against] any survivor” (Moyamba, male).

### Improving the healthcare system

In addition to improving their own situation, a few survivors suggested the need to improve health centres to help the country end the outbreak. In their opinion, health facilities are currently not well equipped, with insufficient drugs and other resources and are difficult to access: “I believe if the health workers have the right skills, facilities and the community have access to the needed resources, it will contribute to moving the country to Ebola free” (Western Area Rural, female). Survivors also recommended improving sanitation, including drinking water, latrines and hand washing.

### Survivors' contribution to the Ebola response

All the survivors considered it their role to help their country end the Ebola outbreak and they should be “used as partners in the Ebola fight” (Kambia, male). A few of them mentioned that their first-hand knowledge of Ebola and their lack of fear due to perceived immunity would support their involvement. They strongly believed that they should become involved in social mobilisation. One survivor explained multiple ways in which Ebola survivors could become active in the fight against Ebola: “We can (…) talk to people in our communities (…). On contact tracing, we survivors can go and meet with people that might have come into contact with Ebola infected persons and [have] ran away and [we can] explain to them how we (…) survived because we got early treatment. We will encourage them to comply with the quarantine” (Western Area Rural, female). Many were already involved in sharing their personal experiences with the community in churches and mosques, restaurants, schools or markets. They reported that they had an important role in Ebola prevention and control, and also that this role could help financially and emotionally sustain them as they adjust to being a survivor. Some also thought that they should be involved in contact tracing and others saw themselves as informal enforcers of Ebola response efforts.

## Discussion

Our findings suggest that survivor experiences, emotions and attitudes changed over time as they moved from disease onset to treatment, discharge and to life post-discharge. Major themes identified across the interviews included acute fear and depression linked to Ebola diagnosis, negative experiences with EHUs, positive experiences with ETUs, feelings of joy and thankfulness on discharge, altruistic motivations for the prevention of Ebola through sexual transmission and concerns about their financial situation and discrimination from their communities.

The findings demonstrate that survivors' knowledge and attitudes about sexual transmission risk largely reflected counselling messages to abstain or use condoms. It should be noted that messaging guidelines on sexual transmission evolved during the outbreak as more information became available which could have led to misperceptions and distrust of information in survivors. It could also explain why interviewed survivors released earlier in the outbreak from ETUs engaged more frequently in sexual activities as survivors were first told to use condoms or abstain for 3 months after their release, which later changed to 6 months and the 12 months. Accurate, evaluated and consistent messages on sexual transmission and appropriate condom use need to be developed and delivered to survivors and their partners. Targeted approaches to HIV and sexually transmitted infection prevention have proved to be highly effective in Sierra Leone and could be applied to the prevention of sexual transmission of Ebola.^17^ One recent study has shown that Ebola virus RNA can persist in semen for 284 days after the disease onset and evidence on viral persistence in other body fluids is still emerging.^18^ ^19^ In light of this new evidence, semen testing and counselling services may help reduce the possibility of resurgence. Having survivors registered and part of a longer term care and support system sustaining community engagement to reduce Ebola exposure risks while minimising stigma of survivors may also reduce future risk.^20^

While all interviewees reported supportive attitudes from family members, about a third faced discrimination and stigma from their communities after their discharge. Continued promotion of the integration and acceptance of Ebola survivors in communities could reduce stigma and improve health seeking behaviours. An example of a successful reintegration strategy is the Firestone Project in Liberia where teams travelled to survivor homes before their discharge to meet with communities and engage them in plans to welcome survivors home.^21^ These types of strategies were replicated in some regions of Sierra Leone and some NGOs, such as Partners in Health, also supported employment opportunities for survivors in the response.^22^ The Sierra Leone Association of Ebola Survivors was created in January 2015 to seek and ensure protection and welfare of its registered members, including Ebola survivors, orphans, widows and widowers.^23^ Such organisations can strengthen the voice of survivors, giving them more power to demand financial and emotional security and support from governments. They can improve the visibility of survivors, help raise awareness about their experiences and struggles, lobby government and international partners for improvements to services offered to survivors, and provide a forum in which survivors can support each other.

The Government of Sierra Leone collaborated with the United Nations Development Programme (UNDP) for the implementation of the ‘Social Rehabilitation and Payment to Ebola Survivors Project’ which aims to prevent conflict and build resilience by addressing social marginalisation and discrimination of Survivors.^24^ Local NGOs, such as Focus1000, and international NGOs, such as Partners in Health (PIH) and Wold Hope International (WHI), have implemented interventions to support survivors. These interventions have already shown that by addressing issues of stigma and discrimination at both the community level and the national level, individual attitudes and actions towards survivors may shift from the need to exclude to the need to include. It is important to maintain and improve these existing activities, as well as to ensure they are implemented to cover all survivors across West Africa. Communication strategies that support survivor reintegration into the community need to be evaluated and further developed.

Interviewees emphasised that the improvement of health services is important for ending Ebola transmission. The West-African Ebola outbreak has shed light on issues related to existing medical and epidemiological capacity to respond to emerging disease outbreaks, including problems with the organisation and performance of health systems.^25^ Furthermore, challenges around building community trust and confidence in the healthcare system persist. Efforts to understand and address trust and confidence in the healthcare systems within particularly heavily affected communities are critical for future planning. This could start with the empowerment and engagement of survivors and entire communities in their health, and ensuring government and international actions are transparent and well communicated to the public.

This study revealed that half of interviewees reported negative experiences in EHUs. These may have been influenced by the fact that they were ill earlier in the outbreak, when services were not yet fully functional and misconceptions about the disease and its transmission where common. These misconceptions, especially when strengthened by fear, might have impacted beliefs, attitudes and behaviours of staff working at EHUs towards Ebola patients. On the other hand, nearly all survivors were positive about their ETU experiences. The difference in experiences at EHUs and ETUs could be explained by the lower staff to patient ratios, poorer pay and lower levels of qualifications of staff in EHUs compared with ETUs. ETUs were restricted to Ebola patients, and had therefore higher levels of expertise and preparation to take care of Ebola patients.^26^ ^27^

Ending an Ebola outbreak evidently requires coordinated efforts between government, public health systems and community structures, including strong involvement from trusted community leaders. Survivors were strongly motivated to help end Ebola and to improve the healthcare system. They could serve as valuable resources in connecting the national Ebola response teams to local communities. Some studies have also shown that survivors have a role to play in engaging communities by teaching others how Ebola is transmitted and by helping families understand the need for isolation of individuals with symptoms.^28^ ^29^ A previous Ebola outbreak saw survivors working alongside safe burial teams, contact tracers and community educators, which, to some extent, also happened in Sierra Leone.^11^ They can potentially contribute to Sierra Leone's readiness to prevent, detect and respond to future outbreaks as well as in strengthening public confidence in the healthcare system. Ways of incorporating survivors into response leadership roles also need to be identified.

Acute fear and depression emerged as a major theme among interviewees when discussing their initial experiences with the disease. Many survivors were still facing substantial emotional challenges, financial difficulties, dealing with having lost family members and decreased normal interactions with community members at the time the interviews were conducted. Coupled with some persistent discrimination, survivors faced myriad economic, social and health challenges. Addressing these concerns could facilitate survivors' reintegration into communities. The medical and social service interventions offered within CPES target these issues.^16^ Emotional challenges faced by survivors could first be addressed by listening to survivors at an individual level (counselling sessions) and a community level (giving them a voice to share their experiences). It is also important to address stigma in communities, by seeking support from community leaders, organising talks and discussions, or publicly supporting survivors in the media. Finally, there could be a benefit from empowering survivors by including them in future outbreak response activities and giving them an active role in preventing Ebola in their communities.^21^ ^30^

More research is needed to identify gaps in the health systems, as well as the education, religion, business and government systems, which may contribute to survivor isolation and stigma. Evaluation of current activities referred to in this discussion will also be important to assess their actual impact on survivors and identify strengths, weaknesses, opportunities and threats. Many of the issues revealed in this evaluation are similar to those identified among survivors from previous Ebola outbreaks;^11^ ^31^ ensuring that lessons learnt from this and previous evaluations are translated into Ebola response, recovery and health protection activities is essential and could help not only Sierra Leone but also Liberia and Guinea.

## Limitations

While this evaluation aimed to provide a detailed understanding of the experiences of Ebola survivors to inform Ebola recovery strategies and preparedness for future outbreaks in Sierra Leone; the findings may not be representative of all survivors in Sierra Leone. The outbreak has already lasted over 17 months and impacted all 14 districts, and some of the attitudes and experiences identified in this evaluation may be specific to their geographic location, the ETUs and EHUs they attended, and time of infection. Additionally, survivors were invited to participate in the interviews through local leaders who may have selected the most aware and informed members of the community and also those who may have had the most positive experiences. Finally, EHUs and ETU experiences of those who did not survive are not captured by these interviews.

## Conclusion

This evaluation provides a description of the diverse experiences of survivors, following many months of intensified Ebola response efforts, and gives an insight into beliefs of survivors about sources of transmission, healthcare seeking behaviours, life at EHUs and ETUs, acceptance by communities and loved ones and understandings of sexual transmission. This study also stresses the importance of empowering survivors and having them contribute to Sierra Leone's preparedness to face future outbreaks. Addressing the myriad and diverse challenges survivors face such as discrimination, stigma, loss of employment and health problems—should form a centre pillar of an Ebola outbreak recovery strategy.

## References

[1] Hewlett BS, Epelboin A, Hewlett BL Medical anthropology and Ebola in Congo: cultural models and humanistic care. Bull Soc Pathol Exot 2005;98:230–6. 16267966

[2] Kunii O, Kita E, Shibuya K [Epidemics and related cultural factors for Ebola hemorrhagic fever in Gabon]. Nihon Koshu Eisei Zasshi 2001;48:853–9. 11725529

[3] WHO Afro. Statement on the end of the Ebola outbreak in Sierra Leone 2015. http://www.afro.who.int/en/sierra-leone/press-materials/item/8140-statement-on-the-end-of-the-ebola-outbreak-in-sierra-leone.html

[4] UK Parliament. Ebola: responses to a public health emergency 2016. http://www.publications.parliament.uk/pa/cm201516/cmselect/cmintdev/338/33804.htm

[5] Sierra Leone National Ebola Response Centre. Lessons learned from the response to the Ebola birus Disease outbreak in Sierra Leone—May 2014 to November 2015 Summary Report. Freetown: Government of Sierra Leone, 2015.

[6] Organization WH. Ebola Situation Report—30 March 2016. http://apps.who.int/ebola/current-situation/ebola-situation-report-30-march-2016

[7] Carod-Artal FJ Post-Ebola virus disease syndrome: what do we know? Expert Rev Anti Infect Ther 2015;13:1185–7. 10.1586/14787210.2015.1079128 26293407

[8] Qureshi AI, Chughtai M, Loua TO Study of Ebola virus disease survivors in Guinea. Clin Infect Dis 2015;61:1035–42. 26060289 10.1093/cid/civ453

[9] Varkey JB, Shantha JG, Crozier I Persistence of Ebola virus in ocular fluid during convalescence. N Engl J Med 2015;372:2423–7. 10.1056/NEJMoa1500306 25950269 PMC4547451

[10] Follow-up Study on Public Knowledge, Attitudes, and Practices Relating to EVD Prevention and Medical Care. (KAP 4 Preliminary Findings) Ministry of Health, Sierra Leone.CDC. Focus1000. http://focus1000.org/index.php/downloads-resources/send/4-ebola-kap-study/32-kap-4-preliminary-findings

[11] Lee-Kwan SH, DeLuca N, Adams M Support services for survivors of Ebola virus disease—Sierra Leone, 2014. MMWR Morb Mortal Wkly Rep 2014;63:1205–6. 25522090 PMC5779533

[12] FOCUS 1000, US Centers for Disease Control and Prevention, UNICEF. Follow-up study on public knowledge, attitudes, and practices relating to Ebola virus disease prevention and medical care in Sierra Leone (Ebola KAP-2), Final Report. Freetown, Sierra Leone: FOCUS 1000, 2014.

[13] FOCUS 1000, US Centers for Disease Control and Prevention, UNICEF. Study on public knowledge, attitudes, and practices relating to Ebola virus disease prevention and medical care in Sierra Leone (Ebola KAP-3), Final Report. Freetown, Sierra Leone: Focus 1000, 2014 http://reliefweb.int/sites/reliefweb.int/files/resources/Ebola-Virus-Disease-National-KAP-Study-Final-Report_-final.pdf

[14] FOCUS 1000, US Centers for Disease Control and Prevention, UNICEF. Follow-up study on public knowledge, attitudes, and practices relating to Ebola virus disease prevention and medical care in Sierra Leone (Ebola KAP-4), Preliminary Report. Freetown, Sierra Leone: FOCUS 1000, 2015.

[15] UNICEF. Sierra Leone Ebola Situation Report, 2015.

[16] World Health Organization. Ebola outbreak to recovery: Sierra Leone progress report. Sierra Leone: WHO, 29 Jan 2015. https://www.unicef.org/appeals/files/UNICEF_Sierra_Leone_EVD_Weekly_SitRep_29_January_2015.pdf

[17] Statistics Sierra Leone, ICF International. Sierra Leone Demographic and Health Survey 2013. Freetown, Sierra Leone. Rockville, Maryland, USA: SSL and ICF International, 2014.

[18] Deen GF, Knust B, Broutet N Ebola RNA persistence in semen of Ebola virus disease survivors – preliminary report. N Engl J Med 2015. doi:10.1056/NEJMoa1511410 10.1056/NEJMoa1511410 PMC5798881 26465681

[19] Mate SE, Kugelman JR, Nyenswah TG Molecular evidence of sexual transmission of Ebola virus. N Engl J Med 2015;373:2448–54. 10.1056/NEJMoa1509773 26465384 PMC4711355

[20] Cheik N The human factor. Bull World Health Organ 2015;93:72–3. 10.2471/BLT.15.030215 25883399 PMC4339971

[21] Arwady MA, Garcia EL, Wollor B Reintegration of Ebola survivors into their communities—Firestone District, Liberia, 2014. MMWR Morb Mortal Wkly Rep 2014;63:1207–9. 25522091 PMC5779529

[22] Partners in Health. Sierra Leone. http://www.pih.org/country/sierra-leone

[23] World Health Organization. WHO meeting on survivors of Ebola virus disease: clinical care of survivors, 3–4 August 2015 Meeting report. Freetown: WHO, 2015.

[24] UNDP. Livelihood skills and mothly stipends for ebola survivors Freetown, 2016 http://www.sl.undp.org/content/sierraleone/en/home/presscenter/pressreleases/2016/06/21/livelihood-skills-and-monthly-stipends-for-ebola-survivors.html

[25] Abramowitz SA, McLean KE, McKune SL Community-centered responses to Ebola in urban Liberia: the view from below. PLoS Negl Trop Dis 2015;9:e0003706 10.1371/journal.pntd.0003706 25856072 PMC4391876

[26] Johnson O, Youkee D, Brown CS Ebola holding units at government hospitals in Sierra Leone: evidence for a flexible and effective model for safe isolation, early treatment initiation, hospital safety and health system functioning. BMJ Glob Health 2016;1:e000030. doi:10.1136/bmjgh-2016-000030 10.1136/bmjgh-2016-000030 PMC5321322 28588922

[27] Barnes S, Hussain N, Hogan J The view from the Ebola Treatment Centre, Makeni, central Sierra Leone. Emerg Med J 2015;32:571–3. 10.1136/emermed-2015-205002 26045442

[28] Stein ZA, Tocco JU, Mantell JE To hasten Ebola containment, mobilize survivors. Int J Epidemiol 2014;43:1679–80. 10.1093/ije/dyu233 25492949 PMC4276067

[29] Epstein JM, Sauer LM, Chelen J Infectious disease: Mobilizing Ebola survivors to curb the epidemic. Nature 2014;516:323–5. 10.1038/516323a 25519116

[30] Marais F, Minkler M, Gibson N A community-engaged infection prevention and control approach to Ebola. Health Promot Int 2016;31:440–9. 10.1093/heapro/dav003 25680362

[31] De Roo A, Ado B, Rose B Survey among survivors of the 1995 Ebola Epidemic in Kikwit, Democratic Republic of Congo: their feelings and experiences. Trop Med Int Health 1998;3:883–5. 10.1046/j.1365-3156.1998.00322.x 9855400

