# Supplementary material for: ‘Once there is life, there is hope’ Ebola survivors' experiences, behaviours and attitudes in Sierra Leone, 2015
Source: BMJ Glob Health. 2016 Nov 18;1(3):e000108. doi: 10.1136/bmjgh-2016-000108 (PMC5321361; doi:10.1136/bmjgh-2016-000108)
Supplement: Supplementary data [file bmjgh-2016-000108supp_appendix.pdf]

APPENDIX 1: IN-DEPTH INTERVIEWS TOPIC GUIDE

1. Please describe what your life was like before you got sick with Ebola
    a. Probe: where did you used to live and who were you living with?
    b. Prove: Please tell me what your day-to-day life was like in your…
        i. Household
        ii. Community
        iii. Public places such as the mosque, church, or school
2. Thanks for sharing with us what your life was like before you got Ebola. Now, if you don't mind, we would like to discuss with you some of the circumstances surrounding how you may have gotten infected
    a. Probe for…
        i. When do you think you got Ebola?
        ii. Where do you think you got Ebola?
        iii. How do you think you got infected?
        iv. Who do you think you might have gotten the disease from?
        v. What signs/symptoms did you have?
3. When you first got sick, what were some of your thoughts?
    a. Probe: How did you feel emotionally?
    b. Probe: How did you feel physically?
4. Please describe the steps that you took in seeking help when you got sick
    a. Probe: From the time you started feeling sick, how long did it take before you first sought help?
    b. Probe: Who did you go to help when you first got sick?
        i. What were the main reasons for seeking help from them first?
    c. Probe: Where else did you seek help?
        i. What were the main reasons for seeking help from them afterwards?
5. For interviewees who delayed medical treatment:
    a. Probe for:
        i. What made you not want to seek medical help?
        ii. {Further probe for cost, distance, and generalized fear, etc.}
6. Please describe the services and facilities you used after you got sick
    a. Based on the responses received, further probe for:
        i. What was your experience with the 117 line/district line?
        ii. What was your experience with the ambulance team?
        iii. What was your experience with other first responders?
        iv. What was your experience in the Holding Centre? (with healthcare workers? With other facility staff?)
        v. What was your experience in the Ebola treatment centre? (with healthcare workers? With other facility staff?)
7. Please describe what your life has been life after you were released from the Treatment Centre
    a. Probe: Where do you now live and who are you living with?
    b. Probe: What has changed since you got Ebola?
    c. Probe: What has remained the same?
8. What does your day-to-day life look like since your release from the treatment centre?
    a. Probe: What has your interaction been with family members and relatives?

   b. Probe: What has your interaction been with your partner/husband/boyfriend/girlfriend?
   c. Probe: What has your interaction been with people in your community?
   d. Probe: What has your interaction been with your Mosque/Church?
9. How have people treated you since your return from the Ebola Treatment Centre?
   a. Probe for…
      i. Family members and relatives?
      ii. Partner/husband/boyfriend/girlfriend?
      iii. Neighbours and other community members?
      iv. Healthcare workers?
      v. Community leaders? (chief, religious leader, Mammy Queen)
   b. Probe: what are the reasons that you think people treat you the way they do?
10. Please describe your understanding of the risk of getting Ebola from sexual transmission
    a. Probe: What sexual experiences have you had since your return from the Ebola Treatment centre?
    b. Probe: How have your sexual relationships changed since before you got sick with Ebola?
    c. Probe: What should people be doing to prevent sexual transmission of EVD?
11. What stories have you shared about your experience since your return from the Ebola Treatment Centre?
    a. Probe for…
       i. Where have you shared these stories of your experience?
       ii. How often have you shared stories of your experience as a survivor?
       iii. Who have you shared these stories with?
12. What opportunities/benefits/services have you received or have available to you as an Ebola Survivor?
    a. Probe: Who is providing these opportunities/benefits/services?
    b. Probe: How long have you been receiving them?
    c. Prove: What has it done for you?
13. What challenges do you currently face as an Ebola survivor?
    a. Probe: what are the sources of these challenges?
    b. Probe: How long have you faced these challenges for?
    c. Probe: How have they impacted your day-to-day life?
14. What can be done to improve the situation for Ebola survivors?
    a. Prove for in the…
       i. Household?
       ii. Community?
       iii. Public places like schools, church/mosque, market?
       iv. Country as a whole?
    b. How do you think this will help move Sierra Leone to becoming Ebola-free?
15. What role do you think Ebola survivors can play in moving Sierra Leone to become Ebola-free – in the household, in the community, in public places like schools/church/mosque/markets, and in the country as a whole?
    a. Probe for the following areas:
       i. Ebola prevention
       ii. Ebola treatment
       iii. Contact tracing
       iv. Social mobilisation

v. Communication
vi. Others?

APPENDIX 2: SCREENING TOOL & QUESTIONNAIRE FOR IN-DEPTH INTERVIEWS WITH EBOLA SURVIVORS

1. Age: ________ (if less than 18 years, DO NOT PROCEED)
2. Government issued certificate confirming Ebola Survivor status:
   a. Available and signed (PROCEED)
   b. Available but not signed (CHECK WITH SUPERVISOR BEFORE PROCEEDING)
   c. Not available (STOP)
3. Gender:
   a. Male
   b. Female
4. Current District:
   a. Western Area
   b. Bombali
   c. Kambia
   d. Moyamba
   e. Port Loko
   f. Other: ____
5. Residential district at time of infection:
   a. Same as current
   b. Different from current; specify: ______________________
6. Has your residence changed between the period you got sick and now?
   a. Yes
   b. No
7. What work were you doing when you got sick with Ebola?
   a. Private business (excluding petty traders)
   b. Plumber / Carpenter / Electrician
   c. Petty Trader
   d. Farmer
   e. Teacher / lecturer / instructor
   f. Public transportation driver (taxi, buses, podapoda)
   g. Okada rider
   h. Medical or health professional
   i. Other Government employee (not already listed above)
   j. Student
   k. Unemployed
   l. Retired
8. What kind of work do you currently do?
   a. Private business (excluding petty traders)
   b. Plumber / Carpenter / Electrician
   c. Petty Trader
   d. Farmer
   e. Teacher / lecturer / instructor
   f. Public transportation driver (taxi, buses, podapoda)
   g. Okada rider
   h. Medical or health care professional

        i. Other Government employee (not already listed above)
        j. Student
        k. Unemployed
        l. Retired
    9. What is you highest level of education?
        a. No formal education
        b. Some primary school
        c. Completed primary school
        d. Completed Junior Secondary School (JSS)
        e. Completed Senior Secondary School (SSS)
        f. Completed Diploma / Postsecondary Training
        g. Completed Bachelors
        h. Completed Masters / Doctorate
        i. No Response
    10. DATE when released from ETU (dd/mm/yyyy): ____________________________
    11. Where did you get your treatment? {insert actual name} ____________________

Post In-depth Interview Survey with Ebola Surveys

1. Who do you currently live with?
    a. Family / relatives
    b. Friends
    c. Neighbours
    d. Live alone
    e. Other(s); specify: ____________________________________
2. Are these the same people you lived with before you got sick?
    a. Yes
    b. No
3. Which of the following best describes you?
    a. Single
    b. Married / have partner
4. Excluding you, how many people got infected with Ebola in the household where you lived? ___________ (If zero, then skip Q5 & Q6)
5. How many people died from Ebola in the household where you lived? _______
6. Excluding you, how people survived Ebola in the household where you lived? ______
7. Which of the following was true for the 30 days' period before you got sick? (read all choices and check all that apply)
    a. Lived in the same household with someone suspected/confirmed to have had Ebola
    b. Took care of a sick person (Go to Q3)
    c. Participated in a funeral/burial (Go to Q2)
    d. I don't know / not sure (Go to Q3)
8. Who did you first seek treatment from? (only select one)
        a. Medical professional (at a health care facility)
        b. Medical professional (outside of the health care facility)
        c. Traditional healer
        d. Spiritual healer
        e. Sowei
        f. Other: _______________________________
9. How long since you first started feeling sick did you seek treatment?
    a. Enter days: _______________
10. Since your release from the Ebola Treatment Centre, how would you rate your day-to-day interaction with community members?
    a. Very good
    b. Good

    c. Not good / not bad
    d. Bad
    e. Very bad

11. Have you ever been sexually active since your release from the Ebola Treatment Centre?
    a. Yes
    b. No
12. During the last time you had sex, did you use a condom?
    a. Yes
    b. No
